# Optical Coherence Tomography Retinal Nerve Fibre Layer and Ganglion Cell Complex Measurements in Normal Southern Nigerian Eyes

**DOI:** 10.7759/cureus.33101

**Published:** 2022-12-29

**Authors:** Osamudiamen C Obasuyi, Ugochukwu E Osuji, Christian O Ifijen, Margaret A Imafidon, Wilson A Ovienria, Irene E Eguaojie, Tessy E Eigbedion, Anita A Alikah

**Affiliations:** 1 Department of Ophthalmology, Irrua Specialist Teaching Hospital, Irrua, NGA

**Keywords:** macular ganglion cell complex, retinal nerve fiber layer thickness, normative data, optical coherence tomography (oct), glaucoma

## Abstract

Introduction

Glaucoma is the leading cause of irreversible blindness worldwide. It is more severe in people with African heritage, and intraocular pressure remains the only modifiable risk factor in managing glaucoma. Attempts to improve the diagnosis and monitoring of glaucoma are ongoing. One of those attempts is the development of optical coherence tomography (OCT). However, there is a theoretical possibility of a delayed or wrong diagnosis of glaucoma using the OCT because of racial, age, and sex differences in the RNFL (retina nerve fibre layer), GCL (ganglion cell layer), and GCL+IPL (ganglion cell layer and inner plexiform layer) thickness.

Objective

This study aims to provide the measurements of RNFL, GCL, and GCL+IPL in normal eyes of southern Nigerian patients and specifically to evaluate the relationship of these measurements to gender, age, intra-eye variability, and the Topcon SD-OCT normative database.

Method

Three hundred and four eyes of 152 patients who had normal OCT scans using the 6x6 RNFL (four sectors) and Macula scans of the Topcon OCT-1 3D Maestro OCT machine were included for analysis. Parametric tests were used to interrogate the relationship between normally distributed parameters and gender, age, and the Topcon reference database. Non-parametric tests were used for non-normally distributed data.

Results

The male-to-female ratio was 1:1, and ages ranged between 18 and 71 for both genders. The average RNFL values were 111.49 ± 10.44 (right eye - RE) and 111.96 ± 9.66 (left eye - LE). For the GCL, average values were 66.23 ± 4.4 (RE) and 66.34 ± 4.19 (LE). GCL+IPL values were 104.02 ± 6.71 (RE) and 103.89 ± 6.66 (LE). There was no difference between genders (X^2^ = 56.467; df = 46; p = 0.160), and RNFL, GCL, and GCL+IPL values showed a significant reduction as the age of the respondents increased. There was a significant difference between RNFL, GCL, and GCL+IPL values and the Topcon reference database, p < 0.001.

Conclusion

Significant differences exist between the Southern Nigerian eyes' RNFL, GCL, and GCL+ IPL values and the Topcon OCT-1 3D Maestro reference database. While randomised control trials and extensive multi-centre studies have not been conducted to determine the possible effects of these differences between measured values and reference databases of the OCTs, they need to be considered while diagnosing and managing glaucoma with the OCT.

## Introduction

The leading cause of irreversible blindness worldwide is glaucoma, responsible for 11% of all blindness worldwide in adults aged 50 and older [1-2]. With the progressive destruction of retinal nerve fibres and retinal ganglion cells, glaucoma slowly causes irreversible blindness. The prevalence of glaucoma varies depending on the nation or region studied, with blacks/Africans presenting with more severe clinical pictures [3].

Currently, intra-ocular pressure remains the only modifiable risk factor for glaucoma, and modulating the intraocular pressure is the current therapeutic goal of available glaucoma care [4]. However, detecting structural damage and monitoring glaucoma progression are still essential in management [5]. Traditionally, the detection of structural damage in glaucoma was done by optic disc photographs. However, optic disc photographs have the limitation of poor inter-observer agreement and do not allow clinicians to quantify change adequately [6].

Optical coherence tomography (OCT) has addressed quantifying structural changes in the retinal nerve fibre layer (RNFL) in glaucoma. Image acquisition in the new generation of OCTs has advanced to the point where scans of 2 mm are now possible [7]. The ability of the OCT to take very thin scans of the retina provides objective, quantifiable estimates and accurately estimates early retinal ganglion cell layer (GCL) and macular ganglion cell complex (GCC) loss. It has, therefore, become the instrument of choice in detecting structural damage and glaucoma progression [7]. By accurately measuring the RNFL, GCL, and macula GCC, the OCT can provide a pre-perimetric diagnosis of glaucoma and guide therapeutic decisions after instituting care by monitoring progression [8].

The ability of the OCT to provide such reproducible and accurate measurements is due to advancements in technology and a strict reliance on normative databases of normal eyes. Unfortunately, these features are also some of its disadvantages. With different proprietary software, image acquisition techniques, and differences in the composition of their respective normative databases, OCT reports are not transferable between the commercially available machines [9-10]. More importantly, the lack of diversity in the normative databases of the OCT machines theoretically poses a problem in the proper interpretation of OCT reports in patients, especially those underrepresented in the normative databases.

While we cannot overstate the importance of normative databases in OCTs, their limitations are apparent. These limitations include the exclusion of children younger than 18 years and a lack of diversity regarding the spread of various ethnic groups [9,11-12]. These limitations have become a point of discussion as multiple reports have shown that the normative databases used in the OCTs show significant ethnic, age, and sex variability compared to other groups. For example, using the Spectralis SD-OCT, Ismail et al. [13] demonstrated that South African eyes had thicker RNFL than the normative database Spectralis SD-OCT employed [13]. Other reports using the Spectralis SD-OCT demonstrated this difference among eyes in India, Nepal, and Brazil [14-16]. Using the Stratus SD-OCT, Sani et al. [17] and Mahmud-Ajeigbe et al. [18] demonstrated that the RNFL thickness in Northern Nigerian eyes was similar to Japanese [19] measurements but thicker than the reports from Italy [20].

Theoretically, an argument exists for the likelihood of misdiagnosing patients based on the differences between the groups not fully represented in the normative databases and the database itself. Therefore, normative measurements of these groups serve as reference points for the future development of OCTs and during clinical judgements in these settings. Unfortunately, these measurements are few or do not exist, especially in Nigeria. While the studies by Sani et al. [17] and Mahmoud-Ajeigbe et al. [18] provided RNFL values in Nigerian eyes using the Stratus SD-OCT, no other study has attempted to document the normative OCT values of Nigerians. To the best of our knowledge, this is the first study in Nigeria to provide normative values of the RNFL, GCL, and GCC of normal Nigerian eyes using the Topcon SD-OCT.

The main aim of this study is to provide measurements of RNFL, GCL, and GCL+IPL in normal eyes of southern Nigerian patients and to evaluate the relationship of these measurements to gender, age, intra-eye variability, and the Topcon SD-OCT normative database.

The following hypothesis will be tested to achieve the study's aim and objectives:

H01 = There is no relationship between intra-eye variability, gender, age, and the RNFL and GCC measurements of southern Nigerian eyes.

HA1 = The RNFL and GCC measurements of southern Nigerian eyes are affected by gender, age, and intra-eye variability.

H02 = There is no difference between the RNFL and GCC measurements of southern Nigerian eyes and the Topcon SD-OCT database.

HA2 = The RNFL and GCC measurements of southern Nigerian eyes differ from the Topcon SD-OCT database.

## Materials and methods

This study was a cross-sectional retrospective study of the clinical data of all patients attending the eye clinic of the Ophthalmology Department in the Irrua Specialist Teaching Hospital who had an OCT examination between September 2019 and September 2022 with the Topcon 3D Maestro SD-OCT. The research team obtained ethical approval from the hospital's Human Research and Ethics Board, and all parts of the research adhered to the tenets of the Helsinki Declaration. The team used only de-identified data from the case notes of the respondents. The hospital numbers collected from the case notes were used only to cross-reference OCT data and were not used during data analysis. There was no minimum sample size for this study. All patients' clinical records that met the inclusion criteria were included in the study [21-23].

Inclusion criteria

Included in the study were all relevant clinical data for patients aged 18 years and older (to allow for proper comparison with the normative database). The recruited patients had uncorrected Snellen visual acuities of 6/18 or better in both eyes, with intraocular pressures between 10 mmHg and 21 mmHg. Furthermore, the team recruited only patients with a minimum of cortical lens opacity or posterior subcapsular cataract grade 1 and below. Also, only the clinical data of patients with no past ocular surgical history and a documented dilated fundoscopy showing normal optic discs and no posterior segment pathology was used in this study.

Before being entered for analysis, the minimum requirement was OCT scan quality of 40% and better (6 × 6 3D disc, 3D macular GCC, and GCC+IPL scans). Furthermore, the B-scan image and RNFL maps were analysed for artefacts and were excluded from analysis if artefacts were present.

Exclusion criteria

Patients with comorbidities like hypertension, diabetes mellitus, sickle cell disease, and neurologic disorders like migraine, seizure disorders, or optic disc disorders were excluded from the study. In addition, patients with ocular conditions capable of altering the thickness of the RNFL or GCC layers, like glaucoma, AMD, vitreoretinal interface disorders, or uveitis, were also excluded.

Data analysis

The research team collected data from the case notes and OCT records of all patients who attended the eye clinic during the review period. Patients' data were screened using their present visual acuity and ocular examination in line with the inclusion and exclusion criteria and cross-referenced against the OCT scans. The OCT data were screened using the OCT signal quality and scan quality guidelines as per the inclusion and exclusion criteria. Patient-de-identified data meeting the inclusion criteria were collected using an Excel sheet (Microsoft® Corp., Redmond, WA) before exporting to IBM SPSS Statistics version 28.0 for further statistical analysis.

The frequencies, mean, and standard deviation were calculated for all data. After that, the data were tested for normality. Appropriate parametric (independent samples T-test, paired samples T-test, one-sample T-test, bi-variate correlation) or non-parametric tests (chi-square, independent sample Mann-Whitney U test, Spearman's correlation, related sample Wilcoxon signed rank test) were used to interrogate relationships between normal or non-normally distributed variables and the Topcon SD-OCT normative database. The confidence level was set at 95%, and p-values less than or equal to 0.05 were considered significant.

## Results

One hundred and fifty-two patients were recruited for this study, and three hundred and four eyes were included for analysis. The male-to-female ratio was 1:1, with a mean age of 37.74 years (±13.17) for females and 39.92 years (±13.40) for males. The oldest respondent was 71 years old for both genders, while the youngest was 18 years for females and 19 years old for males. There was no statistically significant difference between both groups (X^2^ = 56.467; df = 46; p = 0.160). The mean RNFL, GCC, and GCC+IPL thicknesses are described in Table 1.

**Table 1:** Mean RNFL, GCC, and GCC+IPL values RNFL: retina nerve fibre layer, GCL: ganglion cell layer, GCL+IPL: ganglion cell layer + inner plexiform layer, RE: right eye, LE: left eye

	Min	Max	Mean	SD	95% CI	
Right eye						
Superior RNFL (RE)	91.0	176.0	140.73	15.71	138.14	143.15
Inferior RNFL (RE)	85.0	195.0	146.95	15.995	144.45	149.62
Average RNFL (RE)	67.0	134.0	111.49	10.44	109.75	113.08
Superior GCL (RE)	47.0	78.0	66.86	4.67	66.13	67.62
Inferior GCL (RE)	45.0	76.0	65.58	4.39	64.91	66.27
Average GCL (RE)	46.0	77.0	66.23	4.40	65.57	66.96
Superior GCL+IPL (RE)	68.0	119.0	102.99	6.60	102.00	104.04
Inferior GCL+IPL (RE)	66.0	128.0	104.87	7.21	103.67	105.94
Average GCL+IPL (RE)	67.0	122.0	104.02	6.71	102.96	105.04
Left eye						
Superior RNFL (LE)	105.0	185.0	143.38	15.59	140.86	146.02
Inferior RNFL (LE)	105.0	194.0	149.01	15.16	146.54	151.43
Average RNFL (LE)	89.0	139	111.96	9.66	110.36	113.48
Superior GCL (LE)	50.0	79.0	66.99	4.37	66.32	67.70
Inferior GCL (LE)	51.0	77.0	65.57	4.22	64.93	66.21
Average GCL (LE)	51.0	78.0	66.34	4.19	65.69	67.00
Superior GCL+IPL (LE)	63.0	124.0	102.98	6.76	101.97	104.04
Inferior GCL+IPL (LE)	67.0	125.0	104.76	7.10	103.64	105.84
Average GCL+IPL (LE)	65.0	123.0	103.89	6.66	102.80	104.89

Overall, inferior measurements were higher than superior measurements, in keeping with the ISNT rule. Mean RNFL values were 111.49 (±10.44) and 111.96 (±9.66), GCL values were 66.23 (±4.4) and 66.34 (±4.19), and GCC+IPL values were 104.02 (±6.71) and 103.89 (±6.66) for right and left eyes, respectively. Table 2 shows the tests of the normality of the data.

**Table 2:** Tests of normality of data RNFL: retina nerve fibre layer, GCL: ganglion cell layer, GCL+IPL: ganglion cell layer + inner plexiform layer, RE: right eye, LE: left eye

	Shapiro-Wilk
Right eye	
Superior RNFL (RE)	0.054
Inferior RNFL (RE)	0.103
Average RNFL (RE)	0.001
Superior GCL (RE)	0.005
Inferior GCL (RE)	<0.001
Average GCL (RE)	0.002
Superior GCL+IPL (RE)	<0.001
Inferior GCL+IPL (RE)	<0.001
Average GCL+IPL (RE)	<0.001
Left eye	
Superior RNFL (LE)	0.607
Inferior RNFL (LE)	0.179
Average RNFL (LE)	0.484
Superior GCL (LE)	0.034
Inferior GCL (LE)	<0.001
Average GCL (LE)	0.122
Superior GCL+IPL(LE)	<0.001
Inferior GCL+IPL (LE)	<0.001
Average GCL+IPL (LE)	<0.001

None of the data was normally distributed except the RNFL measurement for the RE and LE, respectively. The data transformation was unsuccessful for the remainder of the data. The mean RNFL and GCC thickness aggregated by gender are described in Table 3.

**Table 3:** RNFL, GCL, and GCL+IPL measurements by gender RNFL: retina nerve fibre layer, GCL: ganglion cell layer, GCL+IPL: ganglion cell layer + inner plexiform layer, SD: standard deviation

Sex		Superior RNFL	Inferior RNFL	Average RNFL	Superior GCL	Inferior GCL	Average GCL	Superior GCL+IPL	Inferior GCL+IPL	Average GCL+IPL
Right eye										
Female	Mean	142.15	148.73	112.75	66.32	65.20	65.83	102.78	104.91	103.91
	SD	14.75	15.94	9.26	4.18	3.80	3.82	5.84	6.47	5.95
Male	Mean	139.33	145.18	110.25	67.32	65.88	66.59	103.07	104.68	103.99
	SD	16.58	15.96	11.41	5.13	4.95	4.94	7.40	7.98	7.51
Left eye										
Female	Mean	144.00	149.95	112.56	66.25	64.95	65.62	102.62	104.40	103.55
	SD	14.524	16.04	8.96	3.75	3.66	3.60	5.44	6.32	5.57
Male	Mean	142.76	148.09	111.37	67.63	66.18	66.97	103.21	104.99	104.08
	SD	16.65	14.28	10.34	4.88	4.22	4.67	7.95	7.89	7.68

The mean female RNFL values were slightly higher, while the males had marginally higher GCL and GCL+IPL values. An independent sample T-test was conducted for the RNFL measurements to explore the relationship between gender and RNFL thickness (Table 4).

**Table 4:** Relationship between gender and RNFL thickness RNFL: retina nerve fibre layer, GCL: ganglion cell layer, GCL+IPL: ganglion cell layer + inner plexiform layer, RE: right eye, LE: left eye

	P-value	95% CI	
Right eye			
Superior RNFL (RE)	0.272	−2.23	7.88
Inferior RNFL (RE)	0.174	−1.58	8.68
Average RNFL (RE)	0.142	−0.85	5.84
Left eye			
Superior RNFL (LE)	0.628	−3.79	6.26
Inferior RNFL (LE)	0.454	−3.01	6.74
Average RNFL (LE)	0.451	−1.92	4.30

In addition, an Independent Sample Mann-Whitney U test was done to explore the relationship between Gender and GCL and GCL+IPL thickness, assuming that the distribution of each measurement was the same across categories of Gender (Table 5).

**Table 5:** Relationship between Gender and GCC, GCC+IPL measurements RNFL: retina nerve fibre layer, GCL: ganglion cell layer, GCL+IPL: ganglion cell layer + inner plexiform layer, RE: right eye, LE: left eye

	P-value
Right eye	
Superior GCL (RE)	0.156
Inferior GCL(RE)	0.318
Average GCL (RE)	0.290
Superior GCL+IPL (RE)	0.572
Inferior GCL+IPL (RE)	0.888
Average GCL+IPL (RE)	0.696
Left eye	
Superior GCL (LE)	0.039
Inferior GCL (LE)	0.068
Average GCL (LE)	0.043
Superior GCL+IPL (LE)	0.398
Inferior GCL+IPL (LE)	0.382
Average GCL+IPL (LE)	0.353

From Tables 4 and 5, the superior and average GCL measurements of the left eyes were statistically different across the gender categories (p = 0.039 and 0.043, respectively). There were no differences in the other measurements across genders. A bivariate correlation was done to identify any associations between the age of participants and RNFL (Table 6).

**Table 6:** Relationship between age and RNFL thickness RNFL: retina nerve fibre layer, GCL: ganglion cell layer, GCL+IPL: ganglion cell layer + inner plexiform layer, RE: right eye, LE: left eye

	Pearson’s correlation	p-Value	95% CI	
Right eye				
Age - superior RNFL (RE)	−0.240	0.003	−0.387	−0.072
Age - inferior RNFL (RE)	−0.193	0.018	−0.356	−0.028
Age - average RNFL (RE)	−0.247	0.002	−0.411	−0.070
Left eye				
Age - superior RNFL (LE)	−0.206	0.011	−0.358	−0.054
Age - inferior RNFL (LE)	−0.231	0.004	−0.370	−0.087
Age - average RNFL (LE)	−0.277	<0.001	−0.418	−0.126

Furthermore, Spearman’s correlation was done for GCC and GCC+IPL measurements (Table 7).

**Table 7:** Relationship between age and GCL, GCL+IPL measurements RNFL: retina nerve fibre layer, GCL: ganglion cell layer, GCL+IPL: ganglion cell layer + inner plexiform layer, RE: right eye, LE: left eye

	Spearman’s Rho	pValue	95% CI	
Right eye				
Age - superior GCL (RE)	−0.183	0.024	−0.352	−0.019
Age - inferior GCL (RE)	−0.233	0.004	−0.389	−0.074
Age - average GCL (RE)	−0.208	0.010	−0.375	−0.043
Age - superior GCL+IPL (RE)	−0.205	0.011	−0.371	−0.043
Age - inferior GCL+IPL (RE)	−0.148	0.070	−0.321	0.022
Age - average GCL+IPL (RE)	−0.179	0.027	−0.346	−0.012
Left eye				
Age - superior GCL (LE)	−0.197	0.015	−0.366	−0.30
Age - inferior GCL (LE)	−0.218	0.007	−0.377	−0.050
Age - average GCL (LE)	−0.216	.008	−0.382	−0.040
Age - superior GCL+IPL (LE)	−0.165	0.042	−0.324	−0.002
Age - inferior GCL+IPL (LE)	−0.159	0.051	−0.322	0.017
Age - average GCL+IPL (LE)	−0.179	0.027	−0.341	−0.004

All measured OCT parameters except the Inferior GCL+IPL measurements of both the right and left eyes demonstrated a significant negative correlation with age. A paired samples T-test (RNFL thickness) and related sample Wilcoxon signed rank test (GCL, GCL+IPL) was done to explore intra-eye variability in Table 8.

**Table 8:** Intra eye variability between RNFL, GCC, GCC+IPL RNFL: retina nerve fibre layer, GCL: ganglion cell layer, GCL+IPL: ganglion cell layer + inner plexiform layer, RE: right eye, LE: left eye

OCT parameter	P-value
Superior RNFL (RE)/superior RNFL (LE)	0.007
Inferior RNFL (RE)/inferior RNFL (LE)	0.023
Average RNFL (RE)/average RNFL (LE)	0.311
Superior GCL (RE)/superior GCL (LE)	0.294
Inferior GCL (RE)/inferior GCL (LE)	0.825
Average GCL (RE)/average GCL (LE)	0.580
Superior GCL+IPL (RE)/superior GCL+IPL (LE)	0.695
Inferior GCL+IPL (RE)/inferior GCL+IPL (LE)	0.403
Average GCL+IPL (RE)/average GCL+IPL (LE)	0.171

Excluding the intra-eye differences noted in the superior RNFL measurements (p=0.007), no other intra-eye differences were noted in the analysis. Table 9 compares the measured values against the Topcon OCT-1 3D Maestro/Maestro 2 reference database [24] using a one-sample T-test. Mean RNFL values were thicker than the reference database, while mean GCL and GCL+IPL values were thinner than the reference database.

**Table 9:** Mean sample values compared against the Topcon reference database RNFL: retina nerve fibre layer, GCL: ganglion cell layer, GCL+IPL: ganglion cell layer + inner plexiform layer, RE: right eye, LE: left eye

		Reference mean	Sample mean	p-Value	95% CI	
RNFL	Superior	126.70	140.73 (RE)	<0.001	11.50	16.55
			143.38 (LE)	<0.001	14.17	19.18
	Inferior	136.56	146.95 (RE)	<0.001	7.82	12.96
			149.01 (LE)	<0.001	10.02	14.89
	Average	104.04	111.49 (RE)	<0.001	5.78	9.13
			111.96 (LE)	<0.001	6.37	9.48
GCL	Superior	70.39	66.86 (RE)	<0.001	−4.33	−2.83
			66.99 (LE)	<0.001	−4.16	−2.75
	Inferior	68.19	65.58 (RE)	<0.001​​​​​​​	−3.36	−1.94
			65.56 (LE)	<0.001	−3.30	−1.95
	Average	71.73	66.25 (RE)	<0.001​​​​​​​	−6.22	−4.81
			66.34 (LE)	<0.001	−6.11	−4.76
GCL+IPL	Superior	106.698	102.99(RE)	<0.001	−4.84	−2.71
			102.99 (LE)	<0.001	−4.87	−2.69
	Inferior	105.702	104.87 (RE)	0.125	−2.07	0.26
			104.76 (LE)	0.082	−2.15	0.13
	Average	106.268	104.02 (RE)	<0.001​​​​​​​	−3.76	−1.60
			103.89 (LE)	<0.001​​​​​​​	−0.389	−1.74

## Discussion

The OCT measurements in southern Nigerian eyes differ significantly from the reference database of the Topcon OCT-1 3D Maestro. While the RNFL in southern Nigerian eyes was significantly thicker, the GCL and GCL+IPL were significantly thinner than the reference database. Theoretically, this provides disadvantages. Thicker RNFL values may delay the diagnosis of glaucoma and other neurological disorders such as dementia, Alzheimer's, and Parkinson's, which show a decrease in RNFL thickness with disease onset [24-27]. In addition, the loss of RNFL has been noted to be more rapid in eyes with thicker RNFL [28]. Since glaucoma has been noted to be more severe in persons of African descent, the possibility of delayed diagnosis and rapid loss of RNFL is of concern. Furthermore, thinner GCL and GCL+IPL values may theoretically lead to an earlier diagnosis of glaucoma and possibly a higher incidence of red disease and subsequent overtreatment. With a high number of people accessing healthcare in Nigeria out of pocket, the attendant strain on personal finances from glaucoma care becomes a significant point of concern.

The RNFL values in our report were also thicker than reports from the northern part of the country [17,18]. The reports from northern Nigeria reported significant intra-eye RNFL thickness variability, which was absent in our report [17]. While it may be argued that the relative differences in the composition of respondents, sample sizes, and differences in machines used in the corresponding studies may be responsible for the observed differences in RNFL thickness, it may also be possible that intra-ethnic variabilities exist and deserve some more attention.

Our report showed that the inferior sectors were largely thicker than the superior sectors, in keeping with other reports on the subject from the country [17] and other parts of Africa [13], irrespective of the machine used. All measured values showed a significant reduction in thickness with aging. This was also in keeping with other reports on the subject [13,14,17,18,28,29]. Gender did not show any effect on the thickness of any measured parameter. However, similar to the reports from the northern part of the country [17], which reported higher RNFL values in females, we also report higher RNFL values in females. However, males had higher GCL and GCL+IPL values.

Strengths of this study include the sample size and the equal distribution of male and female participants. This ensured that only statistically significant differences would be captured in the data analysis. Furthermore, comparing our measurements with the official Topcon reference database allows for an objective interpretation of any statistical analysis done.

Limitations of this study include the fact that refractive errors were not considered in the data collection, and a more diverse geographical spread was not possible because the recruitment of the study sample was hospital-based.

## Conclusions

Significant differences exist between southern Nigerian eyes' RNFL, GCL, and GCL+ IPL values and the Topcon OCT-1 3D Maestro reference database. RNFL values were significantly thicker, while GCL and GCL+IPL values were significantly thinner. These findings present an interesting dimension to diagnosing and managing glaucoma in Nigeria. Furthermore, the differences in thickness between reports from other parts of the country and our report point to the possibility of intra-ethnic variabilities in the OCT measurements in Nigerian eyes. While randomised control trials and large multi-centre studies have not been conducted to determine the possible effects of these differences between measured values and OCT reference databases of the OCTs, they need to be considered while making a diagnosis with the OCT especially in Nigeria with diverse ethnic groups and the risk of severe disease.

## References

[1] Sun Y, Chen A, Zou M (2022). Time trends, associations and prevalence of blindness and vision loss due to glaucoma: an analysis of observational data from the Global Burden of Disease Study 2017. BMJ Open.

[2] Tham YC, Li X, Wong TY, Quigley HA, Aung T, Cheng CY (2014). Global prevalence of glaucoma and projections of glaucoma burden through 2040: a systematic review and meta-analysis. Ophthalmology.

[3] Olawoye O, Kizor-Akaraiwe N, Pons J (2022). Clinical characteristics and stage at presentation of glaucoma patients in sub-Saharan Africa. J Glaucoma.

[4] Gordon MO, Beiser JA, Brandt JD (2002). The Ocular Hypertension Treatment Study: baseline factors that predict the onset of primary open-angle glaucoma. Arch Ophthalmol.

[5] Tatham AJ, Medeiros FA (2017). Detecting structural progression in glaucoma with optical coherence tomography. Ophthalmology.

[6] Jampel HD, Friedman D, Quigley H, Vitale S, Miller R, Knezevich F, Ding Y (2009). Agreement among glaucoma specialists in assessing progressive disc changes from photographs in open-angle glaucoma patients. Am J Ophthalmol.

[7] Gabriele ML, Wollstein G, Ishikawa H, Kagemann L, Xu J, Folio LS, Schuman JS (2011). Optical coherence tomography: history, current status, and laboratory work. Invest Ophthalmol Vis Sci.

[8] Leung CK, Cheung CY, Weinreb RN (2009). Retinal nerve fiber layer imaging with spectral-domain optical coherence tomography: a variability and diagnostic performance study. Ophthalmology.

[9] Mehta N, Waheed NK (2020). Diversity in optical coherence tomography normative databases: moving beyond race. Int J Retina Vitreous.

[10] Kim KE, Park KH (2018). Macular imaging by optical coherence tomography in the diagnosis and management of glaucoma. Br J Ophthalmol.

[11] Kiernan D, Hariprasad S (2010). Normative database in SD-OCT: a status report. A comprehensive look at the evolution of OCT software design and database development. Retinal Phys.

[12] Chaglasian M, Fingeret M, Davey PG, Huang WC, Leung D, Ng E, Reisman CA (2018). The development of a reference database with the Topcon 3D OCT-1 Maestro. Clin Ophthalmol.

[13] Ismail S, Ally N, Alli HD (2020). Retinal nerve fibre layer thickness in a normal black South African population. Eye (Lond).

[14] Alasil T, Wang K, Keane PA, Lee H, Baniasadi N, de Boer JF, Chen TC (2013). Analysis of normal retinal nerve fiber layer thickness by age, sex, and race using spectral domain optical coherence tomography. J Glaucoma.

[15] Appukuttan B, Giridhar A, Gopalakrishnan M, Sivaprasad S (2014). Normative spectral domain optical coherence tomography data on macular and retinal nerve fiber layer thickness in Indians. Indian J Ophthalmol.

[16] Thapa M, Khanal S, Shrestha GB, Sharma AK (2014). Retinal nerve fibre layer thickness in a healthy Nepalese population by spectral domain optical coherence tomography. Nepal J Ophthalmol.

[17] Sani RY, Abdu L, Pam V (2016). Retinal nerve fiber layer thickness measurements of normal Northern Nigerian adults using optical coherence tomography. Ann Afr Med.

[18] Mahmud-Ajeigbe F, AbdulRahman H, Rafindadi A, Abah E (2015). Retinal nerve fibre layer measurements in normal eyes in Zaria using optical coherence tomography. Sub-Saharan Afr J Med.

[19] Oshitari T, Hanawa K, Adachi-Usami E (2007). Macular and retinal nerve fiber layer thickness in Japanese measured by Stratus optical coherence tomography. Clin Ophthalmol.

[20] Carpineto P, Ciancaglini M, Aharrh-Gnama A, Cirone D, Mastropasqua L (2005). Custom measurement of retinal nerve fiber layer thickness using STRATUS OCT in normal eyes. Eur J Ophthalmol.

[21] Iverson SM, Feuer WJ, Shi W, Greenfield DS (2014). Frequency of abnormal retinal nerve fibre layer and ganglion cell layer SDOCT scans in healthy eyes and glaucoma suspects in a prospective longitudinal study. Br J Ophthalmol.

[22] Na JH, Sung KR, Baek S (2012). Detection of glaucoma progression by assessment of segmented macular thickness data obtained using spectral domain optical coherence tomography. Invest Ophthalmol Vis Sci.

[23] Na JH, Sung KR, Lee JR, Lee KS, Baek S, Kim HK, Sohn YH (2013). Detection of glaucomatous progression by spectral-domain optical coherence tomography. Ophthalmology.

[24] (2022). Topcon Healthcare. 111 Bauer Drive Oakland, NJ 074362021.

[25] Parisi V, Restuccia R, Fattapposta F, Mina C, Bucci MG, Pierelli F (2001). Morphological and functional retinal impairment in Alzheimer's disease patients. Clin Neurophysiol.

[26] van der Heide FC, Steens IL, Geraets AF (2021). Association of retinal nerve fiber layer thickness, an index of neurodegeneration, with depressive symptoms over time. JAMA Netw Open.

[27] Yu JG, Feng YF, Xiang Y (2014). Retinal nerve fiber layer thickness changes in Parkinson disease: a meta-analysis. PLoS One.

[28] Leung CK, Yu M, Weinreb RN, Ye C, Liu S, Lai G, Lam DS (2012). Retinal nerve fiber layer imaging with spectral-domain optical coherence tomography: a prospective analysis of age-related loss. Ophthalmology.

[29] Mardin CY (2019). [Retinal nerve fiber layer thickness measurements with optical coherence tomography in glaucoma patients and healthy controls]. Klin Monbl Augenheilkd.

